# Residual Language Abilities Correlate With Diagnosis and Prognosis in Patients With Disorders of Consciousness

**DOI:** 10.1002/cns.71176

**Published:** 2026-10-13

**Authors:** Chuan Xu, Jiaxin Gao, Jing Zheng, Jie Yu, Jian Gao, Jingqi Li, Nai Ding, Benyan Luo

**Affiliations:** ^1^ Department of Neurology Sir Run Run Shaw Hospital, School of Medicine, Zhejiang University Hangzhou China; ^2^ Department of Neurology First Affiliated Hospital, School of Medicine, Zhejiang University Hangzhou China; ^3^ Key Laboratory for Biomedical Engineering of Ministry of Education, College of Biomedical Engineering and Instrument Sciences Zhejiang University Hangzhou China; ^4^ Research Center for Advanced Artificial Intelligence Theory, Zhejiang Lab Hangzhou China; ^5^ Department of Rehabilitation Hangzhou Mingzhou Brain Rehabilitation Hospital Hangzhou China

**Keywords:** disorders of consciousness, language abilities, neural tracking

## Abstract

**Background:**

Evaluating residual consciousness and predicting outcomes in unresponsive patients pose significant clinical challenges. Language assessment is crucial for these patients, and language‐based EEG paradigms have shown promise in aiding diagnosis and prognosis in patients with disorders of consciousness (DoC). However, current language‐based EEG paradigms either require higher‐order cognitive functions, thereby misidentifying some conscious patients as unconscious, or rely on automatic processing, which fails to capture residual consciousness in DoC.

**Methods:**

Newly developed, simple phrase sequences combined with neural frequency tagging were used to explore a language paradigm tailored to a critical processing threshold. This paradigm was designed to reflect the dynamic process of consciousness in DoC, while requiring minimal cognitive load.

**Results:**

Our results showed that significant phrase‐level EEG responses were observed in healthy subjects, MCS patients, and UWS patients in three conditions. Neural tracking of phrase structure showed a progressive decrease across three groups (healthy > MCS > UWS). Among the UWS patients, 52.38% (11 out of 21) and among the MCS patients, 45.83% (11 out of 24) exhibited significant ITPC at the syllable rate in at least one of the three conditions. Additionally, 38.10% (8 out of 21) of UWS patients and 33.33% (8 out of 24) of MCS patients demonstrated significant ITPC at the phrase rate. The language paradigm performance in this study was superior to that of other paradigms in detecting the proportion of residual language ability in DoC patients. Furthermore, cortical tracking at the phrase rate significantly correlated with 12‐month outcomes and effectively classified patients according to their 12‐month prognosis.

**Conclusions:**

The simplified language assessment demonstrates strong potential to detect residual language ability in DoC patients and could potentially be used to detect residual consciousness and determine prognosis in unresponsive patients.

## Graphical abstract

The newly developed simple phrase sequences combined with neural frequency tagging were used to explore a language paradigm that was in a critical state and reflected the process of consciousness in DoC. The new simplified language assessment had the potential to detect residual language ability in DoC patients and could potentially be used to detect residual consciousness and determine prognosis in unresponsive patients.

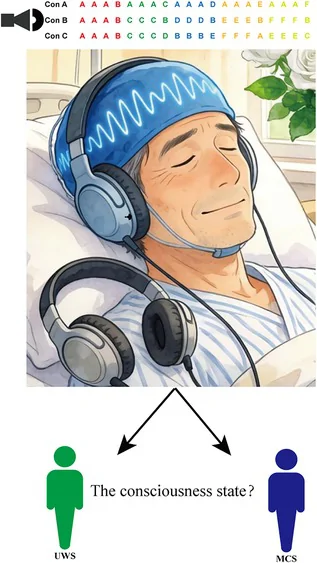

## 1 Introduction

Disorders of consciousness (DoC) result from direct disruptions to the neural systems governing awareness, as well as indirect disruptions to interconnected neural networks [1]. Patients in vegetative states/unresponsive wakefulness syndromes (VS/UWS) and minimally conscious state (MCS) were included in the term DoC [2]. VS/UWS is characterized by wakefulness without awareness [3]. Patients with MCS exhibit inconsistent but reproducible non‐reflexive, cortically mediated behaviors [4]. The distinction between VS/UWS and MCS represents a continuum, reflecting the gradual process of regaining consciousness in severe brain injury. Diagnosing and determining the prognosis of patients with DoC present significant clinical challenges due to the diverse physical and cognitive impairments, fluctuating arousal levels, variable responsiveness to commands, underlying central and peripheral impairments, and the reliance on non‐standardized procedures by clinicians.

The standardized clinical assessment of DoC patients directly evaluates language function across several domains (e.g., the oromotor and communication subscale scores of the Coma Recovery Scale). Comprehension of verbalization, as well as appropriate smiling or crying in response to verbal or visual emotional expression, are considered markers of consciousness [4]. The classification of DoC patients into subgroups, such as MCS without language (MCS−) and MCS with language (MCS+), is directly influenced by the presence or absence of language‐related signs of consciousness. Moreover, language deficits might prevent consistent responses to verbal items, leading to an underestimation of levels of consciousness in DoC patients [5]. Therefore, accurate language assessment is essential for this patient population. Recent studies have introduced language paradigms that elicit rhythmic brain responses corresponding to various speech rhythms (single‐word, phrase, and sentence), indicating that these responses are linked to behavioral assessments of consciousness and can aid in predicting future outcomes for individual patients with disorders of consciousness [6, 7, 8]. Syllable‐level processing engages the bilateral perisylvian region (superior temporal gyrus (STG), supramarginal gyrus (SMG)), left inferior frontal regions (ventral motor cortex (vMC), inferior frontal gyrus (IFG), the anterior insula (AI)), and supplementary motor area (SMA)/pre‐SMA [9]. In contrast, processing at the phrase and sentence levels recruits the superior temporal gyrus (STG), inferior frontal gyrus (IFG), and temporoparietal junction (TPJ) to a greater extent than does syllable‐level processing [10]. These language paradigms demand higher‐order cognitive function and continuous focus, which can lead to an underestimation of consciousness levels in DoC patients with cognitive impairments. Low‐level language processing, such as tracking of the natural speech envelope, may operate automatically without requiring top‐down attention and, as such, may not accurately reflect processes associated with consciousness. It is essential to investigate a language paradigm that critically represents consciousness processes while imposing minimal cognitive demands on the patient.

The neural cortex is attuned to spectrotemporal sound patterns and violations of such patterns induced by rare stimuli embedded in sound streams. According to Taaseh et al., stimuli elicit a more significant response when they are infrequent compared to when they are common [11]. Neurons exhibit sensitivity to sound sequence complexity, with responses to random sequences being equal to or exceeding those to periodic sequences [12]. The mechanism behind these phenomena can be explained by stimulus‐specific adaptation (SSA). Extensive research on SSA has primarily employed the oddball sequence paradigm, typically constructed by selecting tones randomly based on their probabilities [13]. SSA comprises two fundamental processes: repetition suppression and prediction error [14, 15, 16]. As a result of repetition suppression, a lower firing rate is exhibited after every presentation of a standard tone, whereas prediction error indicates an enhanced response upon presentation of a deviant tone [15, 17]. Moreover, the cortical response to oddball sequences varies with changes in the balance between “repetition suppression and deviance detection load” [18]. The ERP local–global auditory paradigm was developed to evaluate brain responses to temporal regularity violations, distinguishing between local (short‐term) and global (over several seconds) effects, and to infer consciousness in DoC patients. The presence of the global effect indicates conscious processing, although it may be absent in conscious individuals unaware of global auditory patterns [19]. However, the cognitive demands of global effects remain excessively high. The ERP local–global paradigm, similar to other language paradigms, exhibits high specificity for conscious processing but low sensitivity.

As mentioned above, the cerebral cortex is sensitive to “adaptation load”, which can be reduced by simplifying sentence structure. Reducing the “adaptation load” may positively affect cortical responses to language stimuli. Therefore, a newly developed simple language paradigm was designed in which the detailed regularities of phrase sequences were varied in each condition, that is, Condition A: The first 3 syllables were identical and relatively certain, whereas the fourth syllables were always different; Condition B: The first 3 syllables were identical and different, whereas the fourth syllables were relatively certain; Condition C: The first 3 syllables and the fourth syllables were always different. Phrases in linguistics consist of words and are important components of sentences, including noun phrases, verb phrases, prepositional phrases, etc. [20]. The “phrases” in this study refer only to chunks of several specific syllables defined in this study, unlike those in linguistics. By simplifying the structure of the phrase stimuli in this study, the cortical responses in DoC were elicited as much as possible to identify the optimal passive language paradigm with the least cognitive demands and the highest accuracy in predicting prognosis and residual language ability in unresponsive patients.

## 2 Methods

### 2.1 Participants

This study included 24 MCS patients (20 males; 48.62 ± 15.23 years) and 21 UWS patients (13 males; 54.24 ± 14.54 years) hospitalized at Hangzhou Mingzhou Brain Rehabilitation Hospital. The inclusion criteria comprised: (i) MCS or UWS diagnosis via Chinese version CRS‐R assessments by DoC experts [2]; (ii) over 1 month post‐brain injury; (iii) age above 18; (iv) no pre‐injury hearing impairment; (v) presence of auditory startle reflex; (vi) absence of centrally acting drugs, neuromuscular blockers, or sedation 24 h prior to the study; (vii) no significant skull bone defects (CT); (viii) CT‐confirmed brain lesions not restricted to the brainstem, excluding locked‐in syndrome. Patients experienced traumatic brain injury, anoxic brain injury, or cerebrovascular disease. Table S1 provides comprehensive patient details, encompassing etiology, diagnosis, CRS‐R outcomes, time post‐injury, and the 12‐month Glasgow Outcome Scale Extended (GOS‐E). There were no significant differences in baseline demographic characteristics, including age, etiological distribution, and brain injury site, between the MCS and UWS groups (Table 1). The Chinese version of the CRS‐R is translated from the original English version and consists of 23 hierarchically arranged items across 6 subscales addressing auditory, visual, motor, oromotor, communication, and arousal processes.

**TABLE 1:** Baseline characteristics of DoC patients.

	MCS	UWS	*p*
Sample size	24	21	
Age (years)	48.63 ± 3.11	54.24 ± 3.17	0.215
Etiology			0.203
TBI (*n*)	17	11	
nTBI (*n*)	7	10	
Lesion type			0.923
Brainstem damage (*n*)	1	1	
Non‐brainstem damage (*n*)	23	20	

Abbreviations: MCS, minimally conscious state; nTBI, non‐traumatic brain injury; TBI, traumatic brain injury; UWS, unresponsive wakefulness syndrome.

The study included 22 age‐matched healthy participants (7 males; 52.14 ± 9.23 years) with no history of neurological disorders. The healthy participants were care workers at Mingzhou Hospital. Participants or their legal guardians provided written informed consent for the experiments in this study.

### 2.2 Behavioral Classification and Outcomes at 12 Months

Patients were divided into MCS and UWS groups based on CRS‐R behavioral assessments. Patient outcomes were assessed using the GOS‐E at 12 months. GOS‐E scores range from 1 to 8, with higher scores indicating better outcomes [21]. Consistent with prior research [22], a threshold GOS‐E score of 3 was used to categorize patients as negative outcome (GOS‐E score ≤ 3) or positive outcome (GOS‐E score > 3).

### 2.3 Stimuli

The stimulus consisted of an isochronous sequence of monosyllables. The auditory sequences contained two linguistic levels: monosyllable and four‐syllable phrases. The four‐syllable phrases were composed of a three‐syllable phrase and a monosyllable word. Monosyllables were presented at a constant rate of 300 ms per syllable, allowing neural tracking of syllables and four‐syllable phrases at different frequencies (3.333 and 0.833 Hz, respectively). As shown in Figure 1, three conditions were presented in the study. In Condition A, the first 3 syllables were identical and relatively certain, while the fourth syllable was always different; in Condition B, the first 3 syllables were identical and different, while the fourth syllable was relatively certain; in Condition C, the first 3 syllables and the fourth syllable were always different. Neuronal responses to a deviant tone are larger compared to a standard tone, a phenomenon known as stimulus‐specific adaptation (SSA). Recent studies have introduced extra control tone sequences to separate the traditional SSA index into two distinct processes: repetition suppression and prediction error. Repetition suppression involves a reduced firing rate with each repeated presentation of a standard tone, while prediction error is indicated by an increased response to a deviant tone. SSA has been studied extensively, primarily using the oddball paradigm. In the oddball paradigm, deviance is typically generated by presenting an infrequent (“deviant”) stimulus amidst a frequent (“standard”) stimulus, with tones selected randomly based on their probabilities. Compared to the oddball sequences, the isochronous sequences in three conditions contained the statistical context of the oddball sequence (including the standard and deviant monosyllable sounds); on the other hand, the “repetition and deviance detection load” could be further modified in the auditory sequences: the first 3 syllables and the fourth syllable were always different or relatively certain. Given the very low level of cognitive function preserved in DoC patients, we sought to maximize the statistical cues of the isochronous sequences to elicit cerebral responses. As shown in Figure S1, there was an upward trend in 0.833‐Hz ITPC in Conditions A, B and C in healthy participants, indicating that the cerebral cortex was more sensitive to the isochronous sequences that involve a greater deviance detection load in Condition C. The sequences were constructed by directly concatenating synthesized monosyllables without pauses. Monosyllables were presented at 3.333 Hz, while phrases were presented at 0.833 Hz, both isochronously.

**FIGURE 1:**
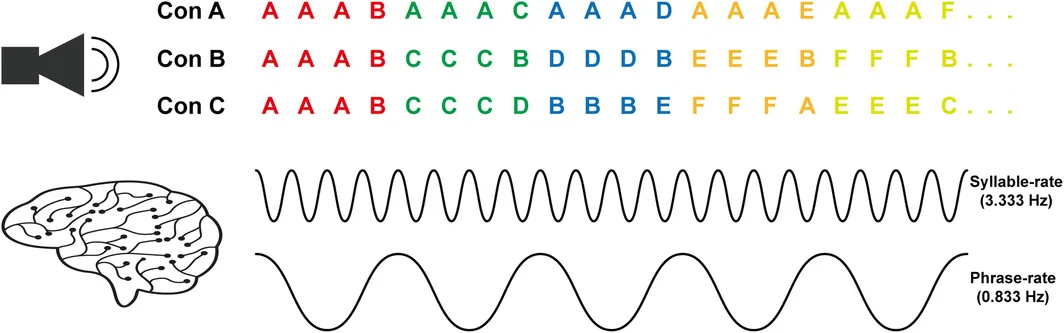
Experimental design. Con A: Condition A; Con B: Condition B; Con C: Condition C. Neural tracking to the rhythms of linguistic structures at different sizes was expected in this study. 0.833 Hz: phrase‐level tracking; 3.333 Hz: syllable‐level tracking.

### 2.4 Procedures

Participants' EEG responses were recorded while they passively listened to isochronous speech. At the start of the experiment, all participants had their eyes open (if they did not open them at the beginning, they could be stimulated with pain to open them). The experiment required healthy subjects to remain still. No other task instructions were given. The study included three conditions. Each condition contained six auditory sequences, and each auditory sequence consisted of 440 syllables (132 s in duration). All 18 auditory sequences in three conditions were presented in a randomized order.

### 2.5 EEG Recording and Preprocessing

EEG signals were captured using a 64‐electrode BrainCap (Brain Products DmbH, Munich, Germany), including an electrode positioned beneath the right eye for electrooculogram (EOG) recording. EEG signals were initially referenced to FCz online and later adjusted offline to the average of the bilateral mastoids. EEG signals were processed using a 12th‐order zero‐phase Butterworth notch filter at 50 Hz to eliminate line noise, an 8th‐order zero‐phase Butterworth low‐pass filter with a 70 Hz cutoff for anti‐aliasing, and an 8th‐order zero‐phase Butterworth high‐pass filter with a 0.3 Hz cutoff to mitigate slow drifts. Signals were sampled at 1 kHz and downsampled to 200 Hz. The least‐squares method was employed to regress EOG artifacts. The EEG signal was processed according to the method outlined by Zou et al. [23]. This study's preprocessing and analyses were conducted using MATLAB software (The MathWorks, Natick, MA).

### 2.6 Frequency‐Domain Analysis

Each condition's trial consisted of a 12‐s EEG data segment. Therefore, each condition consisted of 66 trials. A Discrete Fourier Transform (DFT) was applied to the 12‐s data segment, achieving a frequency resolution of 0.0833 Hz. The ITPC is defined as follows:

$$\begin{array}{c} C\left(f\right)={\left(\sum_{t=1}^{T}\cos\left(\alpha_{\text{ft}}\right)\right)}^{2}+{\left(\sum_{t=1}^{T}\sin\left(\alpha_{\text{ft}}\right)\right)}^{2} \end{array}$$

where *α*_ft_ represents the DFT phase at frequency *f*, and C_*f*_ signifies the inter‐trial phase coherence (ITPC). An elevated ITPC value signifies consistent response phases across trials.

### 2.7 Diagnosis and Prediction Analysis

A three‐class LDA classifier was developed to diagnose healthy controls, MCS, and UWS patients using leave‐one‐subject‐out cross‐validation. The classifier inputs consisted of neural tracking to syllable and phrase structures across three conditions, each characterized by 0.833 and 3.333‐Hz ITPC features. An investigation was conducted to determine whether tracking at the monosyllabic and phrasal levels across the three conditions could predict patient outcomes 12 months following EEG recording. The cross‐validated method previously described for constructing an LDA classifier was employed to distinguish between outcome‐positive (*n* = 21) and outcome‐negative (*n* = 24) patients.

### 2.8 Statistical Analysis

Bias‐corrected and accelerated bootstrap methods were employed for all significance tests [24]. In the bootstrap procedure, all the participants were resampled with replacement 10^4^ times. The peak frequency's response power was compared to the average power of the adjacent four frequency bins, with two bins on each side, using a one‐sided comparison. The significance level was determined by calculating (*N* + 1)/10,001 if the phase coherence at the target frequency exceeds the average coherence of adjacent bins N times in the resampled data.

For unpaired group comparisons, bias‐corrected and accelerated bootstrap methods were employed [24]. Each group's data underwent 10,000 bootstrap resampling iterations. This comparison was one‐sided. The significance level was calculated as (*N* + 1)/10,001 when the mean of one group exceeds (or is less than) that of the other group in *N* out of 10,000 times. This study used Pearson's correlation coefficient (Pearson's r) to evaluate the linear correlation between cortical tracking and the GOS‐E score at 12 months, and FDR correction was applied to the correlation analysis.

Neural tracking to syllable and phrase structure was observed in the results for individual patients across at least one of the three conditions. These 3 variables in three conditions involved multiple comparisons, and the Benjamini‐Hochberg method was used to control FDR [25]. All 3 original *p*‐values were uniformly corrected, with *q* < 0.05 being considered significant after correction.

## 3 Results

The study design involved constructing a hierarchy of linguistic structures of varying sizes, such as syllables and four‐syllable phrases, which were then presented under three experimental conditions to both DoC patients and healthy controls (Figure 1). The ITPC were then analyzed, with 3.333 and 0.833 Hz ITPC reflecting syllable‐level tracking and phrase‐level tracking, respectively. As shown in Figure 2, in Condition A, both syllable‐level neural tracking (3.333 Hz ITPC) and phrase‐level tracking (0.833 Hz ITPC) were significant across the healthy control, MCS, and UWS groups. In Condition B, significant response peaks were also observed at the syllable rate (i.e., 3.333 Hz) and the four‐syllable phrase rate (i.e., 0.833 Hz) in the three groups, that is, healthy group, MCS, and UWS group. Furthermore, ITPC at frequencies harmonically related to the phrase rate (i.e., 1.67 Hz and/or 2.5 Hz) was observed in healthy controls and MCS patients in Conditions A and B. In Condition C, significant syllable‐level neural tracking was observed across healthy, MCS, and UWS groups. In addition, phrase‐level neural tracking was significant in these participants. Response topography revealed that each response peak in healthy individuals, MCS, and UWS patients exhibited a broad spatial distribution, consistently centered near the fronto‐parietal central channels across all conditions.

**FIGURE 2:**
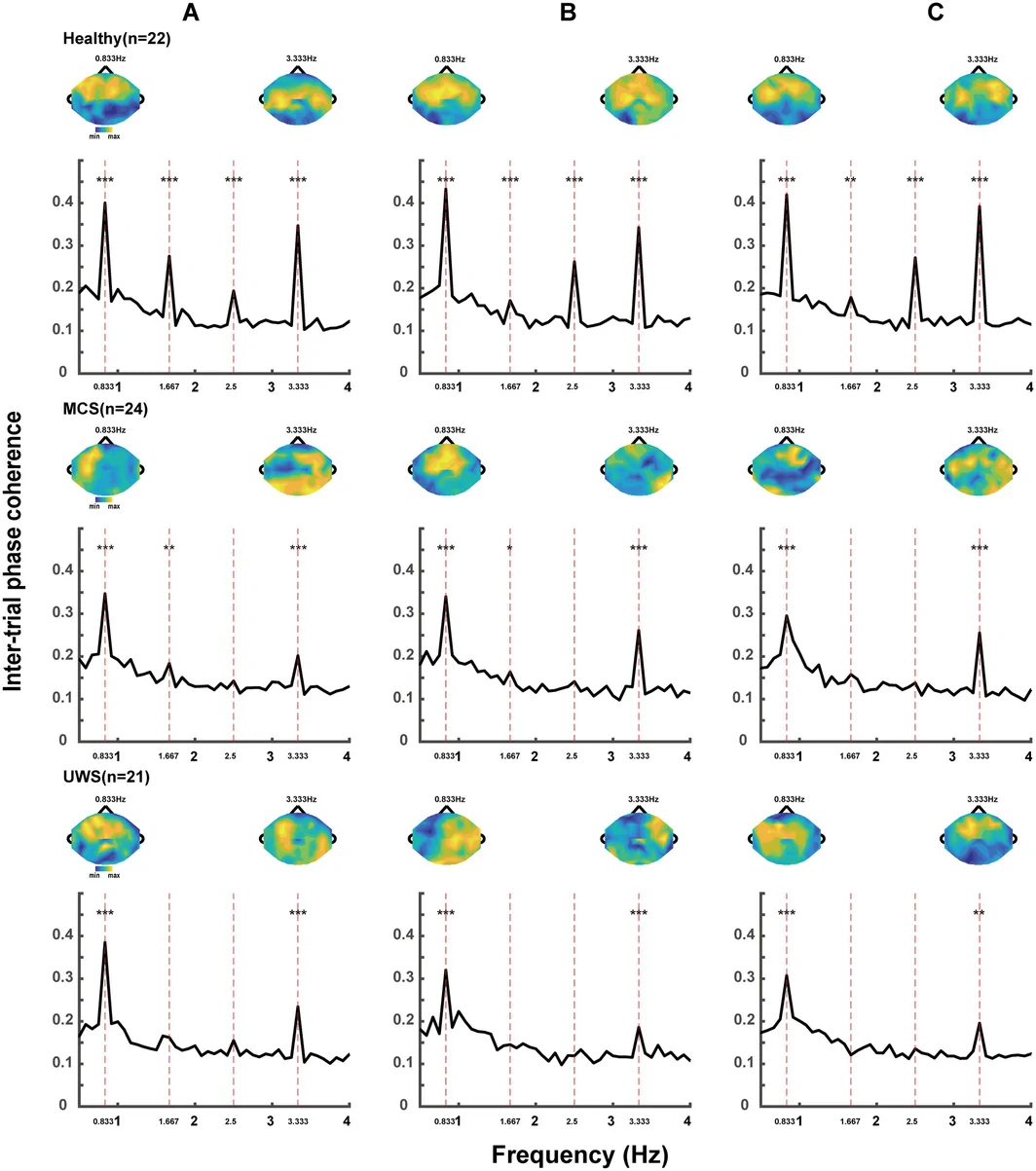
The neural response spectrum and topography in healthy individuals and patients with DoC were analyzed, with significance levels indicated as *p < 0.05, **p < 0.01, and ***p < 0.001 using bootstrap methods. (A) the neural response spectrum & topography in three groups in Condition A; (B) the neural response spectrum & topography in three groups in Condition B; (C) the neural response spectrum & topography in three groups in Condition C. 0.833 Hz: phrase‐level tracking; 3.333 Hz: syllable‐level tracking.

Next, syllable‐ and phrase‐level neural tracking were compared across three conditions among the healthy, MCS, and UWS groups, as shown in Figure 3. In Condition A, MCS and UWS patients exhibited a significant reduction in syllable‐level tracking, as indicated by the 3.333‐Hz ITPC, compared to healthy controls. However, no significant differences were observed in phrase‐level neural tracking, measured by the 0.833‐Hz ITPC, among healthy controls, MCS patients, and UWS patients. In Condition B, MCS and UWS patients exhibited significantly reduced syllable‐level tracking compared to healthy controls, with UWS patients showing a marginally significant reduction compared to MCS patients. Phrase‐level neural tracking was significantly lower in both MCS and UWS patients compared to healthy controls. Although not statistically significant, there was a downward trend in phrase‐rate ITPC between MCS and UWS patients. In Condition C, syllable‐level tracking was significantly reduced in MCS and UWS patients compared to healthy controls, with a marginally significant reduction in UWS patients compared to MCS patients. Besides, neural tracking of phrase structures was significantly lower in both MCS and UWS patients than in healthy controls.

**FIGURE 3:**
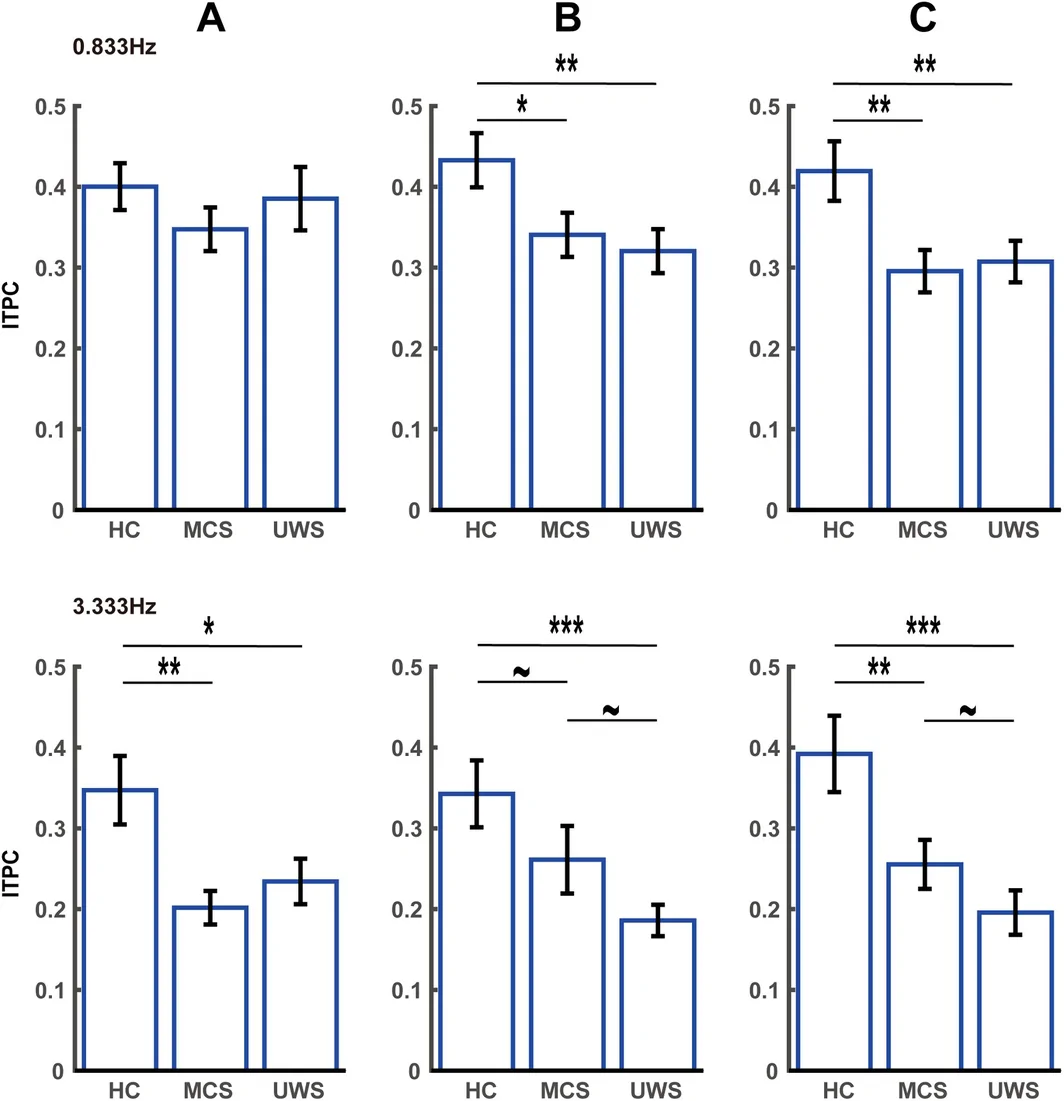
The cortical response between DoC patients and healthy subjects. In Condition A, no significant differences in neural tracking at the phrase level were observed among healthy individuals, MCS patients, and UWS patients. In Condition B, the syllable‐rate ITPC decreased progressively from healthy individuals to MCS patients and then to UWS patients. In Condition C, there was a significant decrease in neural tracking at the syllable level among healthy individuals, MCS patients, and UWS patients. HC, healthy controls; ITPC, inter‐trial phase coherence (*p < 0.05, **p < 0.01, ***p < 0.001, ^**~**^ *p* < 0.1, bootstrap, FDR corrected). 0.833 Hz: phrase‐level tracking; 3.333 Hz: syllable‐level tracking.

Neural tracking to syllable and phrase structure in individual patients in the three conditions, as measured by 3.333‐Hz and 0.833‐Hz ITPC, was shown in Figure 4. 17 out of 22 healthy (77.27%), 11 out of 24 MCS patients (45.83%), and 11 out of 21 UWS patients (52.38%) exhibited significant ITPC at the syllable rate (3.333‐Hz) in at least one of the three conditions (The *p* values represented FDR‐corrected *p* values), indicating the auditory pathway's integrity in these patients (All enrolled patients retained their auditory startle response, as detailed in the inclusion criteria in Methods). Significant ITPC at the phrase rate (0.833‐Hz) was observed in 18 out of 22 healthy (81.82%), eight out of 24 MCS patients (33.33%), and eight out of 21 UWS patients (38.10%). It is shown that integration of syllables into phrase crucially requires top–down attention to participation [26, 27]. And brain responses to passive language and music stimuli are considered as representation of consciousness according to first‐order theories of consciousness [28]. Therefore, phrase‐level tracking in the study may reflect residual consciousness. In fact, eleven (five MCS and six UWS patients) of these 16 (eight MCS and eight UWS patients) (68.75%) patients who had significant ITPC at phrase rate had positive outcomes 1 year after the EEG recordings; 10 patients (8 MCS and 2 UWS patients) of 29 (16 MCS and 13 UWS patients) (34.48%) patients who didn't have significant ITPC at phrase rate had positive outcomes. The proportion of positive outcomes 1 year after the EEG recording in DoC patients who exhibited significant ITPC at phrase rate was significantly greater compared to patients who did not exhibit significant ITPC at phrase rate (68.75% vs. 34.48%, *χ* ^2^: 4.865, *P*: 0.027).

**FIGURE 4:**
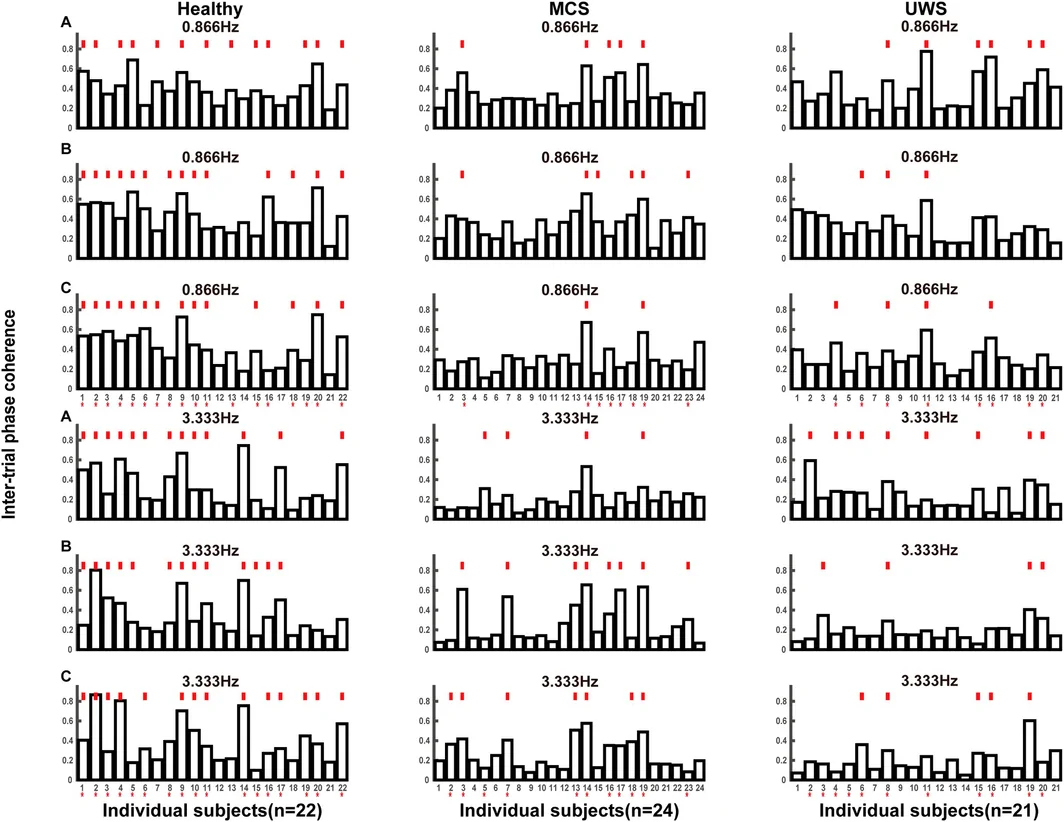
ITPC responses to syllable and phrase structures in healthy and individual patients at three condition levels. ITPC significantly higher than chance in individual patients were marked with red dots at the top of each plot. The red pentagrams at the bottom indicated that significant ITPC at the target frequencies was observed in at least one of the three conditions (The *p* values represented FDR‐corrected *p* values). (A) ITPC responses in individual patients in Condition A; (B) ITPC responses in individual patients in Condition B; (C) ITPC responses in individual patients in Condition C. 0.833 Hz: phrase‐level tracking; 3.333 Hz: syllable‐level tracking.

The correlations between the ITPC across the three conditions and the GOS‐E at 1 year were calculated separately, as shown in Figure 5. Neural tracking at phrase level correlated strongly with GOS‐E at 1 year in Condition A (*r*: 0.646, *p*: 0.00002), whereas syllable‐rate tracking (3.333 Hz) did not correlate significantly with GOS‐E at 1 year. Cortical tracking of phrase and syllable (0.833 + 3.333 Hz) was also correlated strongly with GOS‐E at 1 year in Condition A (*r*: 0.554, *p*: 0.0004). In Condition B, both neural tracking at syllable level and phrase level correlated moderately with GOS‐E at 1 year (*r*: 0.468, *p*: 0.003; r: 0.373, *p*: 0.015, respectively). Furthermore, cortical tracking of phrase and syllable (0.866 + 3.333 Hz) was correlated strongly with GOS‐E at 1 year in Condition B (r: 0.517, *p*: 0.0008). In Condition C, both neural tracking at syllable level and phrase level correlated moderately with GOS‐E at 1 year (*r*: 0.380, *p*: 0.015; *r*: 0.316, *p*: 0.039, respectively). Cortical tracking of phrase and syllable (0.866 + 3.333 Hz) was correlated strongly with GOS‐E at 1 year in Condition C (r: 0.404, *p*: 0.011).

**FIGURE 5:**
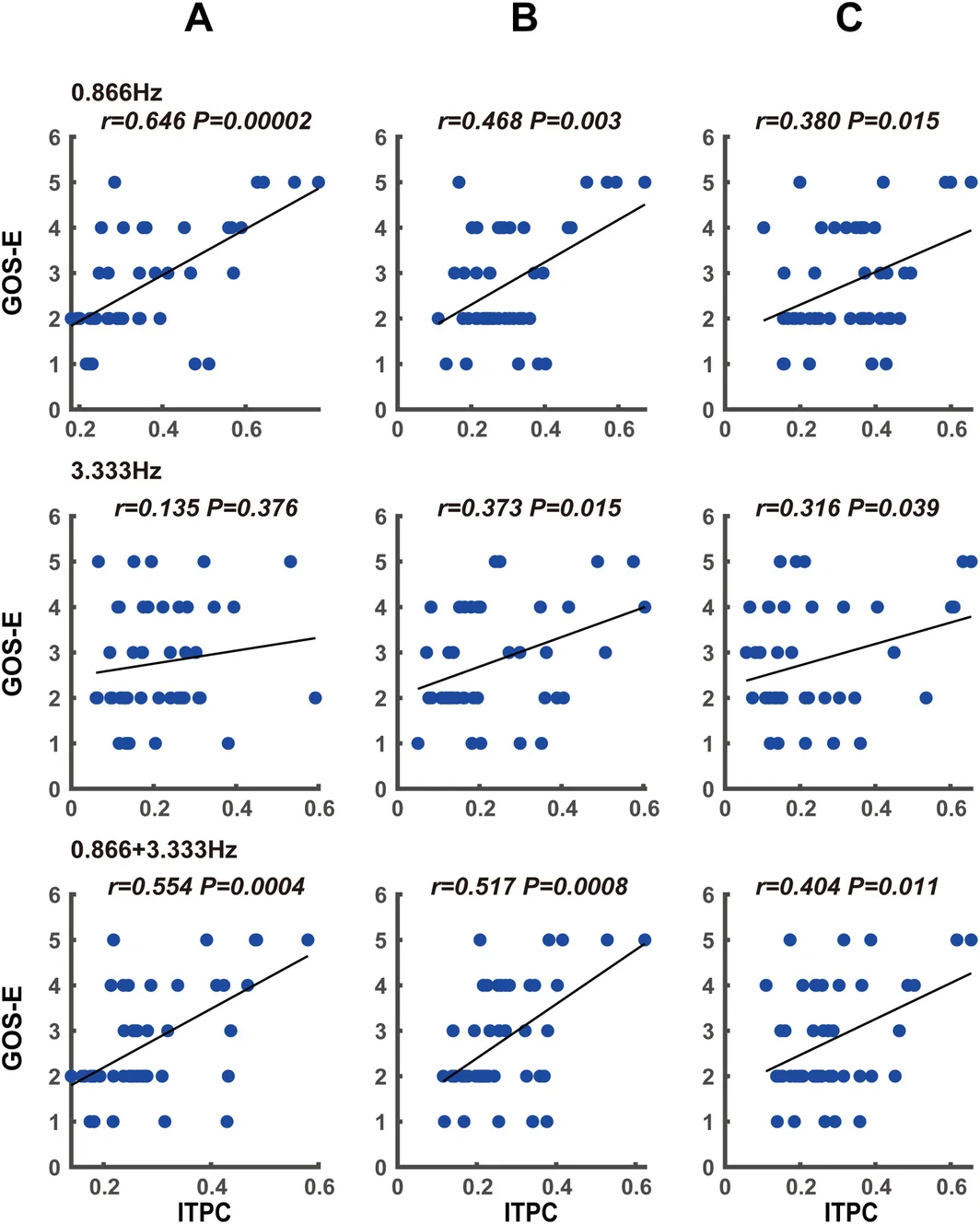
Correlation between cortical tracking and GOS‐E score at 12 months. A strong correlation was found between cortical tracking of syllables and phrases and the GOS‐E score at 12 months. Correlation between cortical tracking and GOS‐E score was analyzed across Conditions A, B, and C. The *p* values represented FDR‐corrected *p* values. 0.833 Hz: phrase‐level tracking; 3.333 Hz: syllable‐level tracking. 0.833 + 3.333 Hz: cortical tracking of phrase and word.

A three‐class LDA classifier was developed to diagnose healthy controls, MCS, and UWS patients using leave‐one‐subject‐out cross‐validation. The classifier inputs included neural tracking of syllable and phrase structures across three conditions (six features: Condition A: 0.833‐Hz and 3.333‐Hz ITPC; Condition B: 0.833‐Hz and 3.333‐Hz ITPC; Condition C: 0.833‐Hz and 3.333‐Hz ITPC). The confusion matrix produced by LDA was shown in Figure 6a. A chi‐squared test was conducted to statistically evaluate the classifier's performance, yielding a significant result (*χ* ^2^ = 10.921, *p* = 0.027, accuracy = 49.3%; see Figure 6a). The decoder achieved diagnostic accuracies of 50% for healthy individuals, 46% for MCS patients, and 52% for UWS patients. Although the diagnostic accuracy of the decoder for healthy, MCS and UWS all significantly exceeded the 33% chance level, its overall performance remained modest, necessitating caution regarding its clinical applicability. Subsequently, the predictive value of syllable‐ and phrase‐level neural tracking across the three experimental conditions was evaluated to determine its utility in predicting one‐year patient outcomes following the EEG recordings. The cross‐validated method previously described for constructing an LDA classifier was employed to distinguish between outcome‐positive (*n* = 14) and negative (*n* = 31) patients. As shown in Figure 6b, the decoder achieved 57% sensitivity and 81% specificity, accurately predicting 8 of 14 outcome‐positive patients and 25 of 31 outcome‐negative patients. Overall, this finding indicated that the decoder demonstrated high clinical value for predicting outcomes of DoC patients.

**FIGURE 6:**
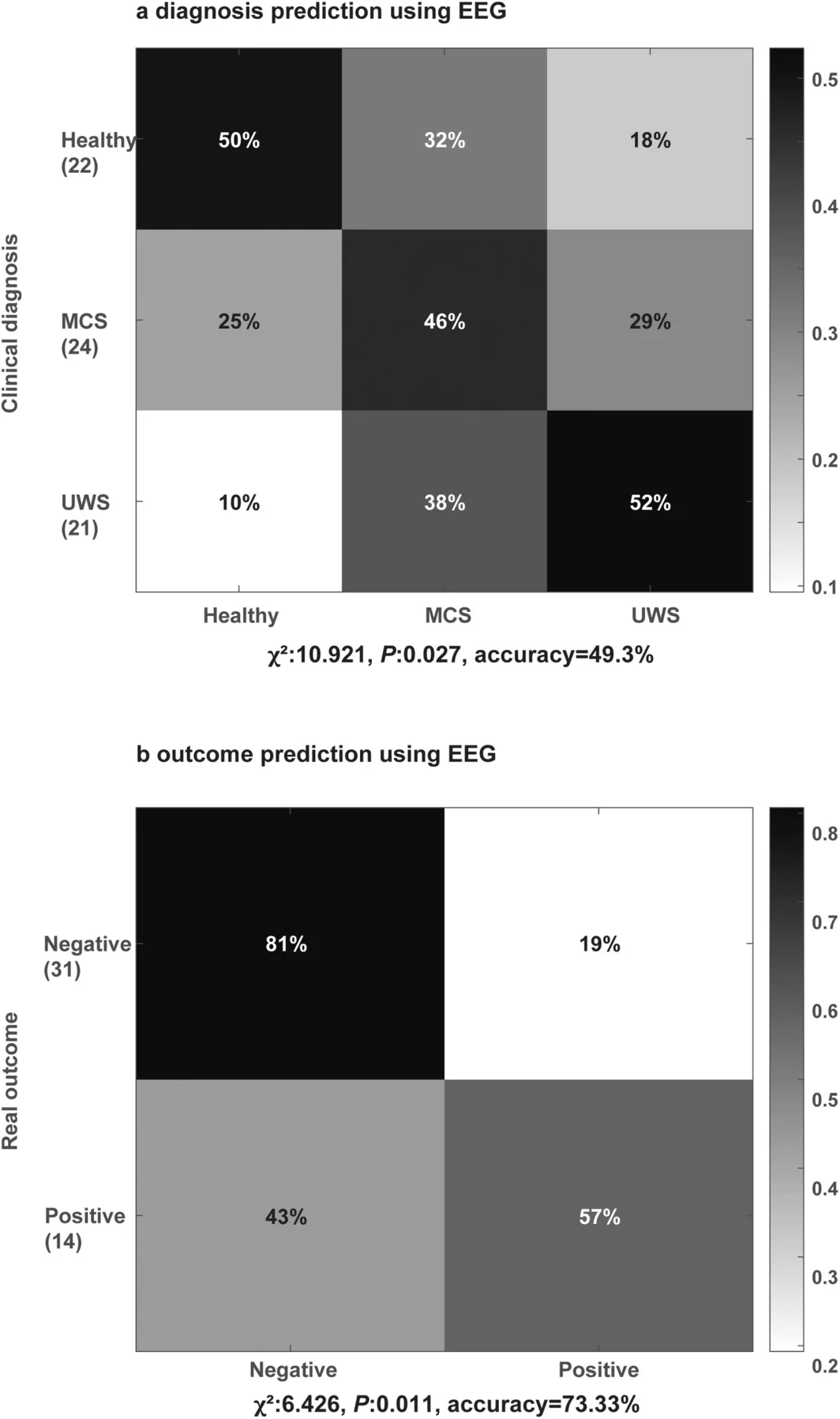
Diagnosis and outcome prediction based on neural tracking to syllable and phrase.

## 4 Discussion

In the present study, a newly developed, simple phrase‐sequence processing paradigm was used to investigate residual language processing ability and level of consciousness in DoC patients. The current study found that DoC patients retained language processing ability, and residual language ability was associated with diagnosis and prognosis.

Patients with DoC encompass neurological syndromes caused by severe brain injuries, including MCS and UWS. VS/UWS is a state of wakefulness without (clinical signs of) awareness [3]. Patients with MCS exhibit inconsistent yet reproducible non‐reflex behaviors mediated by the cortex in response to environmental stimuli [4]. The CRS‐R is typically used in clinical settings to assess levels of consciousness in patients with DoC. Research indicates that behavioral assessments may misclassify up to 40% of non‐communicating patients with DoC as being in a vegetative state or unresponsive wakefulness syndrome (VS/UWS) [29, 30]. Moreover, some UWS patients retain sleep cycles [31]; those with preserved sleep cycles are more sensitive to melatonin intervention and have a better outcome [32]. Accurate diagnosis and prognosis assessment is crucial for family counseling, decision‐making, and rehabilitation program design [33].

Evaluating language ability is a crucial component of the clinical assessment for patients with DoC [34]. The CRS‐R includes six subscales: auditory, visual, motor, oromotor/verbal, communication, and arousal. Language function is not only a subscale of the CRS‐R but also affects other subscales. Thus, deficits in language function may reasonably explain the underestimation of consciousness levels in patients with DoC. A study by Schnakers et al. indicated that 54% of fully conscious patients with aphasia were diagnosed with MCS following the CRS‐R assessment [35]. Indeed, an objective assessment method grounded in language function is crucial for enhancing the evaluation of patients with DoC. Patients with severe brain injury may exhibit higher‐order cortex motor dissociation (HMD), in which brain responses to passive language and music stimuli occur despite a lack of behavioral evidence of language expression and comprehension [36]. Whether UWS patients with HMD should be considered “conscious” remains controversial. First‐order theories of consciousness suggest that consciousness arises from spreading activity in sensory areas [28]. Therefore, HMD would be considered a sign of consciousness. Residual consciousness in patients with prolonged DoC is indicated by the strength of neural responses to language, particularly in tracking higher‐level linguistic structures. These responses correlate with behavioral diagnoses of consciousness and clinical outcomes [6]. Covert language comprehension in patients with acute traumatic brain injury is predictive of recovery from unresponsive states [8]. Aubinet et al. observed that as consciousness levels rose, cortical responses to verbal stimuli intensified, and the resurgence of consciousness post‐coma correlated with the return of both implicit and explicit language processing [37]. In the present study, novel phrase sequences and neural frequency tagging were used to enhance language‐based EEG paradigms for diagnosing and prognosing patients with DoC. Among UWS patients, 11 out of 21 (52.38%) and among MCS patients, 11 out of 24 (45.83%) exhibited significant ITPC at the word rate in ‑at least one condition. Additionally, 8 UWS patients (38.10%) and 8 MCS patients (33.33%) demonstrated significant ITPC at the phrase rate. The language paradigm used in this study outperformed other paradigms in detecting residual language ability among DoC patients.

When listening to continuous speech, cortical activity is concurrently entrained/synchronized to the rhythms of multiple levels of linguistic units including words, phrases, and sentences [10]. It has been established that top‐down attention is required for the neural entrainment to hierarchical language structures, and the speech‐tracking responses were associated with behavioral diagnosis of consciousness in patients with prolonged disorders of consciousness [6, 26]. Neural tracking of chunks could be harnessed to assist in the classification of consciousness and to induce neural plasticity [38, 39, 40]. Furthermore, the cortical tracking of phrases and sentences predicts recovery from acute unresponsive states [8]. However, the natural speech structure may still be too complex for patients with severe brain injury, decreasing the number of patients who may benefit from the speech paradigms. A newly developed simple language paradigm was designed in this study, in which the detailed regularities of phrase sequences were simplified in three conditions. Although not statistically significant, there was an upward trend in 0.833 Hz ITPC in Conditions A, B, and C in healthy participants. Furthermore, the study noted significant phrase‐level tracking across the healthy, MCS, and UWS groups. Residual language abilities in this study were linked to the diagnosis and prognosis of patients with disorders of consciousness. Thus, the simple language paradigm in this study effectively detects consciousness and higher‐order cortical function in patients with DoC.

Some limitations of this study should be acknowledged. The study enrolled 45 patients with DoC, which was a moderate sample size; a larger sample size would be more convincing for research on the diagnosis and prognosis of patients with DoC. The performance of the classifier for diagnosis was mediocre. The UWS patients exhibited numerically higher detection rates than MCS patients at both syllable and phrase levels in the study. The reasons may be that UWS patients include misclassified patients, sampling variability, limited statistical power, and the limited diagnostic performance of the paradigm. There are approximately 40% misdiagnosis cases in DoC reliance on clinical symptoms, and most of the patients who were misclassified may be wrongly classified as being in UWS [2]. Furthermore, the classification classifies 29% of patients diagnosed with MCS in behavioral score as patients with UWS; the awareness of these MCS patients may be underestimated, leading to negative clinical decisions made by their guardians and doctors. In addition, neural tracking at syllable level or phrase level correlated moderately with GOS‐E at 1 year. Even when combined with neural tracking at syllable and phrase level, there was no significant improvement in correlation between neural tracking and outcome at 12 months. This language paradigm should be optimized to improve classifier accuracy, for example, by using more personalized language stimuli and by including patients with DoC of a single etiology to reduce heterogeneity in the future. In addition, the CRS‐R provides a comprehensive assessment of auditory and language‐related functions, which are directly relevant to the auditory EEG paradigm. The CRS‐R is an ideal scale for evaluating patient prognosis in this study, and it should be employed to assess the prognosis of patients with DoC in future auditory paradigms. The gender mismatch among healthy subjects, MCS patients, and UWS patients also affected the study results.

Overall, language processing can remain preserved in DoC patients, and neural representations of linguistic structure offer a valuable paradigm for identifying covert consciousness and informing prognosis in unresponsive individuals.

## Funding

This work was supported by the Natural Science Foundation of Zhejiang Province (LQN25H090004, LQN25H090042), National Natural Science Foundation of China (U22A20293), the National Natural Science Foundation of China (8250050433), and the Major Science and Technology Program of xiaoshan District (2023310).

## Ethics Statement

The study received approval from the Ethics Committees of both the First Affiliated Hospital of Zhejiang University (IIT20200001B) and Hangzhou Mingzhou Brain Rehabilitation Hospital (H2020003).

## Conflicts of Interest

The authors declare no conflicts of interest.

## Supporting information


**Figure S1:** The neural tracking to speech structures between in Condition A, B and C in healthy. ITPC, inter‐trial phase coherence. 0.833 Hz: phrase‐level tracking; 3.333 Hz: syllable‐level tracking.

**Table S1:** The tables listed only the highest score across the 5 CRS‐R assessments. ABI, anoxic brain injury; CRS‐R, Coma Recovery Scale‐Revised; CVD, cerebrovascular disease; MCS, minimally conscious state; UWS, unresponsive wakefulness syndrome; TBI, traumatic brain injury. CRS‐R sub‐scores indicating the assessment of auditory, visual, motor, verbal, communication functions and arousal.

## Data Availability

Data available on request due to privacy/ethical restrictions.
