# Supplementary material for: Residual Language Abilities Correlate With Diagnosis and Prognosis in Patients With Disorders of Consciousness
Source: CNS Neurosci Ther. 2026 Oct 13;32(10):e71176. doi: 10.1002/cns.71176 (PMC13662228; doi:10.1002/cns.71176)
Supplement: Supplementary file 1 [file CNS-32-e71176-s001.docx]

Supplementary Figure.1 **The neural tracking to speech structures between in Condition A, B and C in healthy.** ITPC, inter-trial phase coherence. 0.833 Hz: phrase-level tracking; 3.333 Hz: syllable-level tracking.
