# Supplementary material for: Residual Language Abilities Correlate With Diagnosis and Prognosis in Patients With Disorders of Consciousness
Source: CNS Neurosci Ther. 2026 Oct 13;32(10):e71176. doi: 10.1002/cns.71176 (PMC13662228; doi:10.1002/cns.71176)
Supplement: Supplementary file 2 [file CNS-32-e71176-s002.docx]

| Patient | Clinical diagnosis | Aetiology | Time since  injury (months) | CRS-R  sub-scores | CRS-R total scores | GOS-E at 12months |
| --- | --- | --- | --- | --- | --- | --- |
| 1 | UWS | TBI | 11 | 1-1-1-1-0-1 | 5 | 3 |
| 2 | UWS | TBI | 6 | 1-1-2-1-0-1 | 6 | 2 |
| 3 | UWS | ABI | 3 | 1-0-2-1-0-1 | 5 | 2 |
| 4 | UWS | TBI | 11 | 1-1-2-1-0-2 | 7 | 4 |
| 5 | UWS | CVD | 7 | 1-1-1-1-0-1 | 5 | 2 |
| 6 | UWS | CVD | 8 | 1-1-1-1-0-1 | 5 | 2 |
| 7 | UWS | TBI | 4 | 1-1-1-1-0-1 | 5 | 2 |
| 8 | UWS | CVD | 12 | 1-1-2-1-0-1 | 6 | 1 |
| 9 | UWS | ABI | 13 | 1-1-1-1-0-1 | 5 | 2 |
| 10 | UWS | TBI | 7 | 1-1-1-1-0-1 | 5 | 2 |
| 11 | UWS | TBI | 8 | 1-1-2-0-0-1 | 5 | 5 |
| 12 | UWS | TBI | 3 | 1-0-1-0-0-1 | 3 | 2 |
| 13 | UWS | TBI | 2 | 1-0-2-0-0-1 | 4 | 1 |
| 14 | UWS | TBI | 13 | 1-1-2-1-0-1 | 6 | 1 |
| 15 | UWS | TBI | 4 | 1-1-2-1-0-2 | 7 | 3 |
| 16 | UWS | TBI | 9 | 1-1-2-1-0-1 | 6 | 5 |
| 17 | UWS | ABI | 2 | 1-1-1-1-0-1 | 5 | 2 |
| 18 | UWS | CVD | 4.5 | 1-1-2-1-0-1 | 6 | 2 |
| 19 | UWS | ABI | 2 | 1-1-2-1-0-1 | 6 | 4 |
| 20 | UWS | CVD | 3 | 1-1-2-1-0-1 | 6 | 4 |
| 21 | UWS | TBI | 10 | 1-1-2-1-0-1 | 6 | 3 |
| 22 | MCS | TBI | 2 | 1-2-5-1-0-1 | 10 | 2 |
| 23 | MCS | TBI | 4 | 2-2-3-1-0-2 | 10 | 3 |
| 24 | MCS | TBI | 3 | 1-3-5-1-0-2 | 12 | 4 |
| 25 | MCS | CVD | 10 | 1-3-5-1-0-1 | 11 | 4 |
| 26 | MCS | CVD | 15 | 2-2-5-1-0-2 | 12 | 2 |
| 27 | MCS | TBI | 13 | 1-1-5-1-0-2 | 10 | 5 |
| 28 | MCS | TBI | 10 | 3-4-5-1-1-2 | 16 | 2 |
| 29 | MCS | TBI | 1 | 2-3-5-1-0-2 | 13 | 2 |
| 30 | MCS | CVD | 5 | 2-3-3-1-0-1 | 10 | 2 |
| 31 | MCS | ABI | 12 | 4-5-5-1-0-2 | 17 | 1 |
| 32 | MCS | ABI | 5 | 1-3-1-1-0-2 | 8 | 3 |
| 33 | MCS | ABI | 2 | 1-3-3-1-0-1 | 9 | 2 |
| 34 | MCS | TBI | 8 | 1-1-3-1-0-1 | 7 | 3 |
| 35 | MCS | TBI | 32 | 1-3-3-1-0-2 | 10 | 5 |
| 36 | MCS | TBI | 9 | 1-1-5-1-0-2 | 10 | 3 |
| 37 | MCS | TBI | 6 | 2-3-5-1-0-2 | 13 | 1 |
| 38 | MCS | TBI | 5 | 4-5-5-2-1-2 | 19 | 4 |
| 39 | MCS | TBI | 4 | 2-3-4-1-0-2 | 12 | 2 |
| 40 | MCS | TBI | 10 | 1-3-5-1-0-2 | 12 | 5 |
| 41 | MCS | CVD | 7 | 1-3-5-1-0-2 | 12 | 4 |
| 42 | MCS | TBI | 2 | 1-3-5-1-0-2 | 11 | 2 |
| 43 | MCS | TBI | 4 | 2-3-5-1-0-2 | 13 | 4 |
| 44 | MCS | TBI | 4 | 1-3-2-1-0-2 | 9 | 2 |
| 45 | MCS | TBI | 6 | 2-3-3-1-0-2 | 11 | 4 |

Supplementary Table 1. The tables listed only the highest score across the 5 CRS-R assessments. MCS, minimally conscious state; UWS, unresponsive wakefulness syndrome; CRS-R, Coma Recovery Scale-Revised; ABI, anoxic brain injury; CVD, cerebrovascular disease; and TBI, traumatic brain injury. CRS-R sub-scores indicating the assessment of auditory, visual, motor, verbal, communication functions and arousal.
